# Kynurenic Acid/AhR Signaling at the Junction of Inflammation and Cardiovascular Diseases

**DOI:** 10.3390/ijms25136933

**Published:** 2024-06-25

**Authors:** Alevtina Y. Grishanova, Maria L. Perepechaeva

**Affiliations:** Institute of Molecular Biology and Biophysics, Federal Research Center of Fundamental and Translational Medicine, Timakova Str. 2, Novosibirsk 630060, Russia; aiugrishanova@frcftm.ru

**Keywords:** kynurenine pathway, kynurenic acid, AhR, inflammation, cardiovascular pathology

## Abstract

Persistent systemic chronic inflammatory conditions are linked with many pathologies, including cardiovascular diseases (CVDs), a leading cause of death across the globe. Among various risk factors, one of the new possible contributors to CVDs is the metabolism of essential amino acid tryptophan. Proinflammatory signals promote tryptophan metabolism via the kynurenine (KYN) pathway (KP), thereby resulting in the biosynthesis of several immunomodulatory metabolites whose biological effects are associated with the development of symptoms and progression of various inflammatory diseases. Some participants in the KP are agonists of aryl hydrocarbon receptor (AhR), a central player in a signaling pathway that, along with a regulatory influence on the metabolism of environmental xenobiotics, performs a key immunomodulatory function by triggering various cellular mechanisms with the participation of endogenous ligands to alleviate inflammation. An AhR ligand with moderate affinity is the central metabolite of the KP: KYN; one of the subsequent metabolites of KYN—kynurenic acid (KYNA)—is a more potent ligand of AhR. Understanding the role of AhR pathway-related metabolites of the KP that regulate inflammatory factors in cells of the cardiovascular system is interesting and important for achieving effective treatment of CVDs. The purpose of this review was to summarize the results of studies about the participation of the KP metabolite—KYNA—and of the AhR signaling pathway in the regulation of inflammation in pathological conditions of the heart and blood vessels and about the possible interaction of KYNA with AhR signaling in some CVDs.

## 1. Introduction

Cardiovascular diseases (CVDs) are a leading cause of morbidity and mortality worldwide [1]. The main reason is an insufficient understanding of the pathogenesis of cardiovascular events, indicating the need to identify new factors of the initiation and progression of CVDs. The leading trigger and cause of most CVD is atherosclerosis [2,3]. Modern methods of prevention of CVDs are based on compliance with a healthy lifestyle in combination with pharmacotherapy of traditional risk factors related to dyslipidemia, obesity, and hypertension [4].

The pathophysiology of CVDs is critically linked to the process of inflammation [5], in which immune and other cells secrete excessive amounts of proinflammatory cytokines that exert their actions not only on immune cells but also on many nonimmune tissues and organs, including the heart and blood vessels [6,7]. Metabolites of essential amino acid tryptophan (Trp) are possible factors mediating inflammation in CVDs, and immunologically active metabolites of the kynurenine (KYN) pathway (KP) are especially important in this regard [8,9,10].

The central metabolite of the KP is KYN, a pluripotent mediator and a crucial intermediate in the biosynthesis of many metabolites of the KP that participate in inflammation, immunomodulation, neurological reactions, metabolism, and carcinogenesis [11,12,13,14,15]. KYN performs some of its physiological functions in the central nervous system through the N-methyl-D-aspartate receptor (NMDAR) and α7-nicotinic acetylcholine receptor (a7nAChR), whereas in peripheral tissues, its main mediators are G protein-coupled receptor 35 (GPR35) and aryl hydrocarbon receptor (AhR) [16,17].

AhR is a ligand-activated transcription factor with multiple roles in health and disease and acts by binding to many xeno- and endobiotics in the context of physiological and immune responses [18,19]. AhR is constitutively expressed in hepatocytes, epithelial cells of barrier tissues, cardiac and vascular cells, and in various types of innate-immunity cells (macrophages, dendritic cells, and innate lymphoid cells of type 3 [ILC3 cells]) as well as in the adaptive immune system (T helper [Th] 17 cells and Th22 cells) [20].

AhR plays an important part in the maintenance of the function, health, and physiological homeostasis of cardiac cells and tissues [21]. AhR signaling contributes to the development of cardiovascular pathologies by inducing the expression of proinflammatory interleukins (ILs) IL-1β and IL-8 as well as TNF [22]. In response to inflammatory stimuli, ligand-activated AhR prevents excessive induction of proinflammatory cytokines in cells such as fibroblasts, endothelial cells, and macrophages [23,24,25].

It is known that either abnormally high activation of AhR by high-affinity xenobiotics or its insufficient activation is related to the pathophysiology of CVDs [26,27,28,29,30,31,32]. 

One of the mechanisms underlying the conversion of Trp into an AhR ligand is the metabolism of Trp into KYN by enzymes indoleamine 2,3-dioxygenase (IDO) and tryptophan 2,3-dioxygenase (TDO) [33,34]. IDO becomes activated by various inflammatory stimuli, thereby enhancing KYN metabolism [35]. The activity of IDO and the KP depends on a balance between pro- and anti-inflammatory signals [15], whereas an unbalanced KP may be a cause of various inflammation-related diseases, including CVDs [8,9].

The KP of Trp degradation, which proceeds along three different pathways—the 3-hydroxykynurenine branch, anthranilic acid branch, and kynurenic acid (KYNA) branch—leads to the production of other AhR ligands. Among them, KYNA is a ligand of AhR with a stronger affinity as compared to KYN.

There is almost no research on the health effects of the exogenous KYNA that enters the body [36,37].

The search for new potential therapeutic approaches to inflammatory processes in CVDs is an actively developing research field that is necessary for the successful prevention and treatment of CVDs [38]. From this standpoint, further research steps are needed to fully understand the relevance of KP metabolites—ligands of the AhR—to the modulation of inflammation risk in CVDs and to elucidate the associated molecular mechanisms of action.

In this review, by taking into account the involvement of KP metabolites and AhR in inflammatory responses and the modulation of innate and adaptive immunity, we describe current knowledge about the processes that imply the commonality of AhR with the KP (with an emphasis on its metabolite, KYNA) in the development of some CVDs.

## 2. The Kynurenine Pathway

Essential amino acid Trp undergoes catabolism through several pathways, producing bioactive metabolites that have substantial effects on physiological processes [39].

In mammals, Trp metabolism is divided into three main pathways. The metabolic pathway responsible for most Trp catabolism (95%) is the KP, which metabolizes Trp to nicotinamide adenine dinucleotide. Two other cascades—the indole pathway and serotonin/melatonin pathway—account for 5% and 1–2% of products of Trp degradation [40,41,42,43,44].

**Figure 1:**
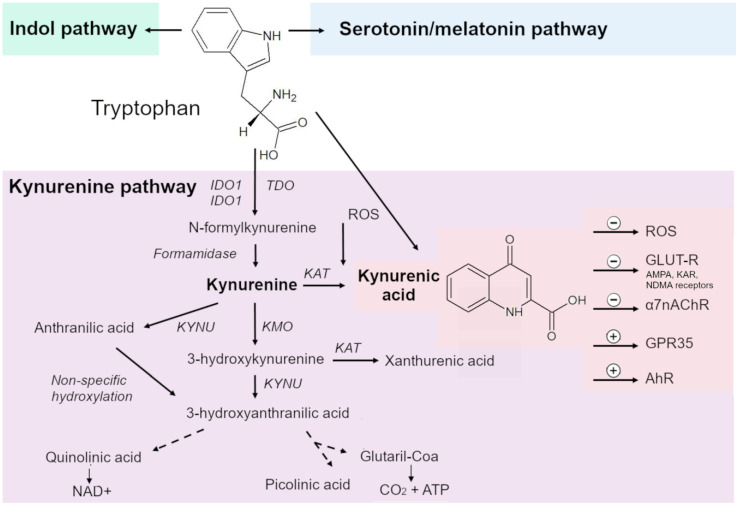
The kynurenine pathway (KP), along with indole and serotonin pathways, is the major cascade of tryptophan (Trp) metabolism. The KP consists of several enzymatic stages, at each of which biologically active metabolites are formed. The central metabolite of the KP is kynurenine (KYN). Rate-limiting enzymes of the KP are indoleamine 2,3-dioxygenase (IDO) 1/2 and tryptophan 2,3-dioxygenase (TDO), which convert Trp into formylkynurenine, which is converted to KYN by kynurenine formidase. From KYN, under the action of a kynurenine aminotransferase (KAT), kynurenic acid (KYNA) is synthesized; under the action of kynureninase, anthranilic acid; and under the action of kynurenine-3-monooxygenase, 3-hydroxykynurenine. During further transformations, 3-hydroxyanthranilic, xanthurenic, quinolinic, nicotinic, and picolinic acids are formed; quinolinic acid is an endogenous source of nicotinamide and nicotinamide adenine dinucleotide (NAD^+^).

The KP consists of several enzymatic steps, each of which produces biologically active metabolites [42] and—in addition to creating endogenous reserves of nicotinamide adenine dinucleotide—participates in several biological processes such as immune regulation, cancer, and inflammation [45,46,47] (Figure 1).

The first and rate-limiting enzymes of the KP are IDO1, IDO2 and TDO, which convert Trp into formylkynurenine. 

Trp is preferentially converted to KYN. These enzymes have different patterns of expression in tissues. TDO is predominantly expressed in the liver and IDO in other organs [15].

IDO1 is expressed in various organs throughout the body and in various immune cells throughout the body, in particular dendritic cells, monocytes, and macrophages. IDO2 is more selectively expressed in antigen-presenting dendritic cells, the liver, kidneys, and the brain [48,49] and does not seem to have a considerable effect on peripheral KYN concentrations [50]. 

Formylkynurenine is converted to KYN by kynurenine formamidase. KYN is processed by three different enzymes. The product of enzymatic transamination of KYN by kynurenine aminotransferase (KAT) isoenzymes is KYNA. In addition, under the influence of kynureninase and kynurenine-3-monooxygenase, KYN is transformed into anthranilic acid and 3-hydroxykynurenine, respectively [15,51,52,53,54].

Further transformations give 3-hydroxyanthranilic, xanthurenic, quinolinic, nicotinic, and picolinic acids [51,52,53,54]. 3-Hydroxyanthranilic acid is a free-radical generator, while quinolinic acid is a known neurotoxin and gliotoxin. Quinolinic acid is an endogenous source of nicotinamide and nicotinamide adenine dinucleotide (NAD^+^).

Implications of activation of the KP depend on which biologically active metabolites are synthesized: KYN, KYNA, quinolinic, or anthranilic acid, which mediate various immuno- and neuromodulatory processes.

### Biological Effects of KYNA

The molecular structure of KYNA, which is one of the metabolites of Trp, has been known since the early 20th century, and metabolic steps of its production from Trp were identified in the first half of the 20th century [55]. 

Under physiological conditions, KYNA is formed by irreversible transamination of KYN, either by a KAT or under the action of reactive oxygen species [56,57,58,59]. 

KYNA is synthesized in many cell types: endothelial and epithelial cells, fibroblasts, pancreatic islet cells, human peripheral blood mononuclear cells, red blood cells, and skeletal muscle cells [60,61,62,63,64,65,66,67]. 

In mammals, four KAT proteins (I–IV) have been described, which are active in various tissues and have relatively weak affinity for their substrate; the main factor controlling the rate of formation of KYNA and its concentration at a site of biosynthesis is the availability of KYN [55]. The functioning of the KAT system in human cardiac muscle has specific features such as optimal pH, cosubstrate specificity, and sensitivity to inhibition by amino acids [58]. In the human heart, KAT I is active and is capable of synthesizing KYNA at low concentrations of KYN [68].

At present, it is thought that KYNA is not metabolized and is one of the end products of the KP. According to the literature, no metabolic pathways for KYNA inactivation have been identified in the biological systems that have been studied [69]. On the other hand, some studies suggest that KYNA can be metabolized to quinaldic acid [70], a compound that has proven antiproliferative activity in vitro against cancer cells [71] and controls synoviocyte viability [72]. There are no data in the literature on the influence of quinaldic acid on the cardiovascular system. 

The first known biological effects of KYNA were related to its function as an endogenous antagonist of NMDA receptors and as a neuroprotector [43,55,73,74,75]. KYNA is an antagonist of all three ionotropic glutamate receptors, including the NMDA receptor, α-amino-3-hydroxy-5-methyl-4-isoxazolepropionic acid receptors, and kainate receptors [76]. In the past two decades, numerous studies have been conducted on the involvement of KYNA in diseases of the central nervous system: schizophrenia, depressive disorder, and Alzheimer’s disease [75]. Besides, KYNA inhibits α7nAChR [55,77].

To date, the connection of KYNA with inflammation and the immune system functioning has been proven [58,78,79]. The anti-inflammatory and immunosuppressive functions of KYNA make possible its role both in the resolution of inflammation and in the creation of an immunosuppressive environment, including one conducive to tumor growth. The immunomodulatory properties of KYNA may be implemented via signaling through GPR35 and pathways mediated by AhR [58,80,81,82]. The effects of KYNA depend on whether the context is inflammatory or homeostatic [58]. 

KYNA is an agonist of GPR35, which is mostly present in the gastrointestinal tract [83]; this feature has sparked research interest in the effects of KYNA on the digestive system and on a number of gastrointestinal pathologies [83]. Signaling via the GPR35 receptor-mediated pathway is also linked with the immunomodulatory effects of KYNA [58].

Along with several other Trp metabolites, KYNA is an endogenous ligand of AhR [76,84,85,86] and is described as a stronger and stabler ligand of AhR than KYN [58]. Signaling through the AhR-mediated pathway is associated with the immunomodulatory actions of KYNA [58].

There is some evidence that KYNA may be a ligand for hydroxycarboxylic acid receptor 3 (HCAR3) and α-2B adrenergic receptor (ADRA2B), but this notion is still a hypothesis [69,87]. KYNA also acts as a free-radical scavenger and antioxidant [76].

The action of KYNA as an immunomodulator under homeostatic noninflammatory conditions has different directions in different cell lines [58]. For instance, KYNA does not affect the secretion of IL-6 or tumor necrosis factor (TNF) by BV-2 mouse microglial cells [88] but reduces the expression of IL-6 mRNA in rat RBL-2H3 mast cells with a subsequent return to baseline [89] and induces IL-6 expression in breast cancer MCF-7 cells [81] and the secretion of TNF, IL-6, IL-1β, and IL-10 by primary mouse splenocytes [90]. KYNA can activate several subpopulations of leukocytes by triggering GPR35, which is expressed in immune tissues, especially in human peripheral monocytes. This property has been demonstrated in an in vitro vascular blood flow model, where KYNA caused the firm arrest of these cells through both β1 and β2 integrin-mediated adhesion; suppression of GPR35 attenuated the kynurenic acid-induced adhesion of monocytic cells, whereas other Trp metabolites that do not bind to GPR35 did not trigger such adhesion processes [91].

In the context of inflammation, Trp metabolism through the KP plays a critical role in the modulation of immune responses [92,93,94]. The process of inflammation is linked with an increase in the concentration of metabolites of the KP [95]. Proinflammatory cytokines shift the metabolism of Trp toward the KP by increasing the expression of IDO. IDO is activated primarily through a proinflammatory biomolecule called interferon-gamma receptor (IFN-γR) [96] as well as via IFN-γR–independent pathways, such as Toll-like receptor 4 (TLR4) [97] or synergistic activation of TLR4, IL-1 receptor beta [98,99], and tumor necrosis factor receptor (TNFR) [99,100]. 

Elevated concentrations of KYNA in the blood have been experimentally induced by various inflammatory conditions in animal models [101,102]. Such inflammatory mediators as IL-1β, TNF, and interferon-γ (IFN-γ) mediate the activation of the KP through induction of the JAK–STAT signaling cascade [95,103,104]. 

The effects of KYNA on immune cells are more uniform during inflammation than under homeostatic conditions. KYNA has anti-inflammatory actions due to its ability to inhibit the differentiation of CD4-positive T cells into the Th17 phenotype and to suppress the release of TNF, IL-4, and IL-23 from activated monocytes [58,105,106]. 

In different types of leukocytes, KYNA has been found to attenuate inflammation caused by various stimuli [e.g., lipopolysaccharide (LPS)] by interacting with GPR35 [58,78,80,82,107]. KYNA weakens LPS-induced expression of IL-23 in mouse dendritic cells and suppresses the differentiation of mouse Th17 cells in vitro by inhibiting GPR35 [108]. 

In ex vivo leukocytes of mice treated with KYNA, the release of TNF in response to the administration of LPS is attenuated [102]. KYNA dose-dependently weakens LPS-induced secretion of TNF in human peripheral blood mononuclear cells expressing the KYNA receptor: GPR35 [82,109]. KYNA attenuates an LPS-induced increase in concentrations of TNF and nitric oxide (NO) in the serum of mice and reduces their LPS-induced mortality [55]. KYNA reduces the secretion of the HMGB1 protein by human peripheral blood monocytes and U937 monocytes, as well as the secretion of α-defensin (human neutrophil peptide 1–3) in whole blood and cultured human granulocytes [109]. KYNA diminishes the release of IL-4 by human-invariant natural killer T cells expressing GPR35 [80].

KYNA suppresses the production of IL-1β and caspase-1 activation in macrophages by specifically inhibiting canonical and noncanonical activation of the NLRP3 inflammasome. By means of GPR35, KYNA reduces calcium mobilization, thereby leading to decreased mitochondrial damage and lower production of mitochondrial reactive oxygen species, thus blocking the assembly and activation of NLRP3 [107]. By blocking NLRP3, KYNA prevents systemic LPS-induced inflammation, sodium urate–caused peritoneal inflammation, and high-fat-diet-induced metabolic disorders [107]. 

It has been shown that KYN metabolism can be shifted toward greater production of KYNA because of overexpression of a KAT in skeletal muscle as a result of physical exercise [110]. Individuals performing endurance exercises show increased expression of KAT mRNAs and proteins as compared to untrained individuals [66,110]. Regular endurance exercise may be a source of KYNA upregulation in the body. In contrast to high-intensity eccentric exercise, aerobic exercise elevates the KYNA concentration in blood plasma within an hour after the exercise [110]. 

It has been proven that KYNA is absorbed from the digestive tract after oral administration [37,70]. The source of KYNA are vegetables, especially cauliflower, potatoes, and broccoli [111]; relatively large amounts of KYNA are found in fermented foods such as kefir and yogurt [112] as well as in spices such as basil and thyme [69]. A large amount of KYNA is present in honey and apiary products; the greatest (very large) concentration of KYNA (2114.9 μg/g) has been found in chestnut honey [113]. Among medicinal herbs, the highest levels of KYNA have been detected in leaves of peppermint, nettle, birch, and horsetail [114].

To date, only one small study has addressed the impact of exogenous KYNA on human health; investigators determined the pharmacokinetic parameters of KYNA after ingestion of chestnut honey and could not detect any adverse effects [37]. In animal studies, administration of KYNA with drinking water has been shown to have antioxidant [115] and immunomodulatory [90] effects in mice, as well as to decrease heart rate in spontaneously hypertensive rats [36]. In experiments on mice, KYNA (5 mg/kg per day) in a high-fat diet slowed the growth of body weight and average daily energy intake; in serum, KYNA diminished triglyceride levels, raised high-density lipoprotein cholesterol levels, slowed an increase in low-density lipoprotein cholesterol levels, and reduced a coronary artery risk index and atherosclerosis index [116]. In other experiments on mice, feeding of chestnut honey (16 weeks, 45 mg of honey ingested per day per mouse) during a high-fat diet attenuated obesity/high-fat-diet–associated insulin resistance and neurodegeneration, improved glucose metabolism, prevented changes in the expression of apoptosis-related genes, and altered blood concentrations of leptin and adiponectin [117].

It has been hypothesized that KYNA, which is responsible for the observed effects in the animal experiments, may be a therapeutic target in human metabolic syndrome, including its manifestations such as heart failure, atherosclerosis, insulin resistance, steatohepatitis, and chronic inflammation [69,118].

## 3. AhR Signaling Pathway

AhR belongs to the bHLH/PAS (basic helix-loop-helix/Per-Arnt-Sim) family of nuclear receptors and is a ligand-activated transcription factor [119,120]. 

Structurally, at its N terminus, AhR, as a representative of bHLH/PAS proteins, has a bHLH domain containing a basic region responsible for DNA binding and a helix-loop-helix region mediating protein–protein dimerization. A nuclear localization signal and a nuclear translocation signal are also located at the N terminus of AhR. Closer to the C terminus of the bHLH domain, a PAS domain is located: subdomain PAS-A is involved in dimerization with AhR nuclear transporter protein (ARNT), whereas subdomain PAS-B takes part in the binding to a ligand. The C-terminal domain is responsible for the interaction of AhR with coactivators and corepressors through three transactivation domains and one inhibitory domain [86,121,122]. 

In the absence of a ligand, inactive AhR is localized to the cytoplasm in a complex with two molecules of heat shock protein 90 (Hsp90) (which stabilize AhR in a conformation with a strong affinity for its ligands), with cochaperone p23 (protecting AhR from ubiquitination and degradation [123], with the XAP2 protein from the immunophilin family (synonyms: AIP and ARA9) (which stabilizes the interaction between AhR and HSP90), and with tyrosine kinase Src [124,125,126].

**Figure 2:**
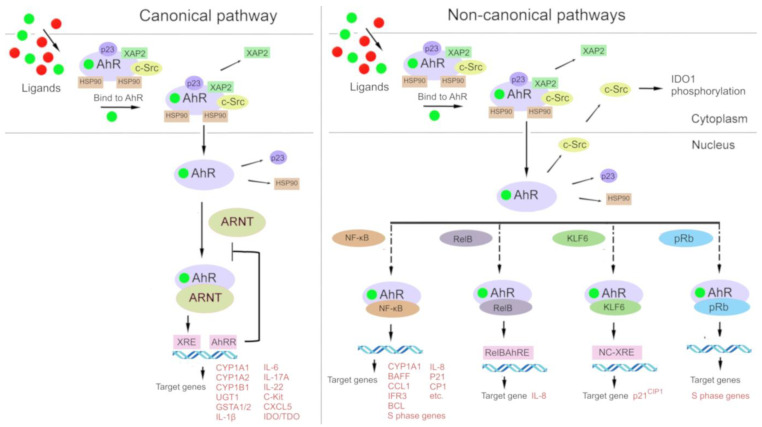
Under physiological conditions, aryl hydrocarbon receptor (AhR) is localized to the cytosol and forms a complex with chaperone proteins: hepatitis B virus X-associated protein 2 (XAP-2), heat shock protein 90 (HSP90), and cytosolic tyrosine-protein kinase Src (c-Src). After binding to a ligand, AhR changes its conformation and moves to the nucleus, where it dimerizes either with AhR nuclear transporter (ARNT) (in the canonical pathway) or with other partner proteins: Krüppel-like factor 6 (KLF6) and transcription factors [nuclear factor-kappa B (NF-κB), an NF-κB subunit known as RelB, and retinoblastoma protein (pRB)]. The AhR–ARNT complex binds to a xenobiotic response element (XRE) in DNA and induces transcription of AhR-responsive genes. Proteins AhR and KLF6 form a heterodimer that recognizes a novel nonconsensus XRE (NC-XRE) and induces the transcription of genes taking part in cell cycle regulation. The AhR–RelB heterodimer recognizes the RelB–XRE complex and induces the transcription of some chemokine genes. The AhR–NF-κB heterodimer induces the expression of inflammation-related cytokines and chemokines. The AhR–pRb heterodimer promotes cell cycle arrest.

In the classic mechanism (Figure 2), after cytosolic AhR binds to a ligand, the complex disintegrates, and the ligand-bound AhR relocates into the nucleus, where it forms a complex with ARNT [86,121]. The resulting AhR–ARNT heterodimer binds to xenobiotic-responsive elements (XREs, consensus sequence 5′-TNGCGTG-3′) in DNA, which perform an enhancer function. After signal transmission from an enhancer to a promoter, transcription of target genes is launched, most of which are related to the biotransformation of xenobiotics (phase I enzymes: CYP1A1, CYP1A2, and CYP1B1; and phase II enzymes: UGT1, GSTA1/2, and ABCG2) [122,127,128]. 

Furthermore, there is a so-called noncanonical transcriptional regulatory pathway involving AhR, where ligand-bound AhR heterodimerizes not with ARNT but with other partner proteins such as Krüppel-like factor 6 (KLF6) and RelB [129,130]. Interactions of AhR with other signal transduction cascades have been described, e.g., with the Keap1/Nrf2/ARE system [131,132,133] and the nuclear factor-kappa B (NF-κB) signaling pathway (Vogel, Wu et al. 2013, Jackson, Joshi et al. 2015, Zhang, Lenardo et al. 2017). 

Direct interaction of AhR with transcription factors signal transducer and activator of transcription (STAT)1 and NF-κB (or with its family members RelA and RelB) can cause inflammatory reactions [134,135,136,137].

Since the discovery of the first genes whose expression is affected by AhR, new target genes have been identified (e.g., inflammatory biomolecules, cytokines, Trp pathway enzymes, and poly[ADP-ribosyl]transferases) that regulate a variety of cellular functions [138,139].

The target genes are involved in AhR repression (e.g., *AhRR*), antioxidant functions (*NRF2*), antimicrobial defense (NADPH oxidase/P40 phox), in organ development (*p21^Cip1^*, *p27^KIP1^*, *p40^phox^*, and *Blimp1*), in immunity and inflammation (*c-Kit*, *IL1B*, *IL6*, *IL17A*, *IL22*, *CXCL5*, and *IDO/TDO*), in reproduction (*CYP19*), and in energy homeostasis (*TiPARP*, *CD36*, and *CD38*) [138].

An additional mechanism of AhR signaling is participation in signal transduction by components of the AhR chaperone complex, which are induced by AhR agonists. In particular, a signal transduction partner called protein tyrosine kinase pp60src, which is released after ligand binding, phosphorylates a wide range of target proteins, including IDO1 [140,141].

For instance, during inflammation, pp60src interacts with epithelial growth factor receptor (EGFR), thereby activating the mitogen-activated protein kinase (MAPK) cascade. This event stimulates the transcription of cyclooxygenase-2 (*COX2*) and matrix metalloproteinase (*MMP*)-1 genes [142].

Initially, AhR was characterized as a receptor of 2,3,7,8-tetrachlorodibenzo-p-dioxin (TCDD) and of polycyclic aromatic hydrocarbons, and its main biological role was thought to be involvement in the detoxification/toxification of various xenobiotics/environmental pollutants [143,144,145,146]. Being a sensor of TCDD and of polycyclic aromatic hydrocarbon-like planar chemical compounds, AhR is a transcriptional activator of several target genes whose enzymatic products have toxic, mutagenic, and carcinogenic properties [144,147,148]. In this capacity, AhR has long been a subject of toxicological research [144,147], and the respective xenobiotics have been named as classic ligands of AhR [149].

Later, numerous exogenous ligands of AhR have been identified that have other physicochemical properties [18,144,146,150,151,152] as well as endogenous ligands of AhR that mediate physiological functions of AhR and its participation in many aspects of cellular activity, either physiological or pathological.

Among the endogenous ligands of AhR, there are tetrapyrroles, heme metabolites, arachidonic-acid metabolites, estrogen, and Trp metabolites such as KYN and KYNA [58,86,153,154,155,156,157]. 

AhR is involved in normal development, immune function, physiological processes in stem cells, differentiation, proliferation, adhesion, and migration of cells [119,136,138,146,158,159,160,161,162,163,164,165,166].

AhR can mediate either pro- or anti-inflammatory processes depending on a ligand bound to it and a cellular context [167]. The proinflammatory effect of TCDD—a “classic” ligand of AhR—and the effects of other dioxin-like chemicals may be mediated by the nongenomic signaling of AhR [138,168]. For instance, TCDD induces a quick increase in intracellular calcium concentration and enzymatic activation of cytosolic phospholipase A2 and cyclooxygenase 2, thereby leading to the accumulation of inflammatory mediators [168]. 

The impact of AhR on inflammation may depend on a transcriptional regulation pathway in the context of its genomic signaling via target genes [18,20,144,169,170], among which there is one “canonical” pathway (where a ligand–AhR complex heterodimerizes with ARNT) and several “noncanonical” ones [where the ligand–AhR complex heterodimerizes with other protein partners, such as Krüppel-like factor 6 (KLF6) and RelB] [129,130]. Besides, AhR can interact with other signal transduction pathways, such as Nrf2 [132] and NF-κB [171,172,173]; AhR signaling is associated with the activity and function of estrogen receptor [18,119]; a ligand–AhR complex can function as a coactivator of E2F1 [129,174]. 

Additionally, AhR is activated by a large variety of endogenous and exogenous ligands [32,175,176], which can—depending on the cellular conditions—play the role of agonists or antagonists of the transcription of AhR-dependent genes. Furthermore, different ligands of AhR can give rise to different transcriptomic profiles within a single cell type, and a single ligand of AhR can induce dissimilar transcriptomic profiles in different cell types [177,178,179]. 

Activation of AhR by various ligands, the participation of AhR in different signaling pathways, and differences in conditions (including a cell type, tissue-specific microenvironment, and the presence of coregulators) can be implemented via different scenarios of changes in the activities of AhR-dependent genes and the resulting influence on the homeostasis of tissues and organs [147,177,178,179,180]. 

The combination of the diverse ligands, transcriptional regulatory pathways, and other molecular interactions yields contradictory results: under some conditions, AhR activation promotes inflammation, whereas under others, AhR activation prevents inflammation. In this article, we focus on the relation between AhR-dependent inflammation and the KP metabolite KYNA within the cardiovascular system.

### 3.1. AhR as a Mediator of Inflammation

The relation between AhR and inflammatory events has been documented in various experimental models [126]. Inflammatory markers such as COX2, TNF, MMPs, early growth response protein 1 (EGR1), prostaglandin (PG) E2, microsomal PGE2 synthase (mPGE2S), NF-κB (RelB and RelA), inducible nitric oxide synthase (iNOS), and IL-8 are associated with AhR signal transduction [126].

#### 3.1.1. AhR-Mediated Control of Cytokine and Chemokine Expression

Some cytokines and chemokines are encoded by target genes of AhR. In keratinocytes, these are the neutrophil attractant chemokine (CXCL5) [181] and IL-1β [182], and in cultured human fibroblast-like synoviocytes, these are IL-1β and IL-6 [183]. Most cells of the immune system, including macrophages, neutrophil granulocytes, and mast cells of the innate immune system, express AhR, as do B cells, many subtypes of T cells, and dendritic cells of the adaptive immune system [184]; in this context, AhR controls the expression of several cytokines.

In Th17 cells, AhR stimulates the production of IL-17A, IL-17F, IL-10, and IL-22 [185,186,187]. IL-17 serves as an inflammation mediator and is essential for host defense against extracellular bacterial and fungal infections, but it also plays an important part in chronic inflammation and in the pathogenesis of autoimmune diseases [188]. AhR activation induces immunomodulatory cytokine IL-10, which suppresses an inflammatory response [187]; IL-22, which is a member of the IL-10 family of cytokines, is also primarily produced by Th17 cells under the influence of AhR and is crucial for protection from gram-negative bacteria as well as participates in the progression and pathogenesis of autoimmune diseases [189]. 

AhR activation can also lead to suppression of inflammatory responses along with a decrease in the amounts of IFNγ, IL-6, IL-12, TNF, and IL-7 [190]. The classic ligand of AhR, TCDD, is capable of inducing the expression of IL-8 in human U937 macrophages through an interaction of signaling pathways of AhR and of NF-κB [191,192].

Endogenous AhR has been found to attenuate the IFN-I response during infection with various viruses, and this phenomenon is reported to be mediated by the endogenous AhR ligand KYNA [193]. Evidence for this effect has been obtained by means of wild-type and AhR^−/−^ mouse embryonic fibroblasts. In addition, in AhR^−/−^ mice, a virus enhances the production of IFN-β, thus limiting viral replication [193]. 

AhR agonists—TCDD, indeno pyrene, and 6-formylindolo[3,2-b]carbazole (FICZ)—have been shown to induce inflammation mediators: TNF, granulocyte colony-stimulating factor, and *MMP*s MIP-1α, MIP-1β, and MIP-2. A study on the expression of *MMP*s in mouse macrophages indicates that AhR-activated macrophages create an inflammatory microenvironment that promotes the progression of pulmonary epithelial-cell fibrosis [194].

AhR plays a key role in the modulation of T-cell differentiation [105], particularly in the differentiation of Th17 cells and regulatory T cells (Treg cells) [185,186,195]. In addition, AhR maintains a balance between Treg cells, including Tr1 cells, CD4^+^CD25^+^Foxp3^+^ Treg cells, and Th17 cells [196]. AhR activation shifts immune-cell differentiation toward Treg cells, thereby ensuring an immunosuppressive microenvironment [197]. Attenuation of an immune response may be beneficial in autoimmune diseases [51] but detrimental in inflammation and cancer [33,198].

In general, although AhR is expressed in several types of immune cells, outcomes of AhR activation by specific ligands may vary [135]. These differences may be explained by the presence of AhR partners other than ARNT in different cell types.

#### 3.1.2. AhR- and NF-κB-Mediated Inflammation

The NF-κB signaling cascade is an important regulator of immune responses and actively interacts with other signal transduction pathways, including AhR-dependent signaling [171,172,173,199]. AhR signaling may promote activation of NF-κB through interactions of subunits RelA and RelB with AhR [200,201,202].

The RelB–AhR dimer binds to a response element called RelBAhRE on the *IL8* promoter, thus linking two signaling pathways to activate transcription of the *IL8* gene. RelB–AhR complexes also target the NF-κB–binding sites that are predominantly recognized by RelB/p52 as well as XREs: a consensus sequence for the AhR–ARNT dimer and consensus sequences of NF-κB [192].

AhR and NF-κB may participate in regulatory feedback loops important for immune regulation. NF-κB induces the expression of the AhR, but AhR can regulate NF-κB signaling [137,192]. In experiments on *AhR*-null mice (*AhR*^−/−^), it has been demonstrated that AhR can control macrophage polarization by regulating NF-κB, whereas AhR depletion enhances NF-κB–dependent macrophage activation in vitro [203]. AhR may participate in the NF-κB signaling pathways that are launched by classic inducers such as LPS [192].

By maintaining the expression of RelB, the expression of AhR limits the proinflammatory production of COX2 and of a PG under the influence of cigarette smoke in mice [24]. In another paper, however, treatment of human lung fibroblasts with AhR antagonists in the presence of a cigarette smoke extract inhibited the nuclear translocation of AhR and the production of COX2, mPGES-1, and PGE [204].

The interaction of AhR with the NF-κB subunit RelA results in the induction of the *c-Myc* gene in mammary cells and can promote cell proliferation and tumorigenesis in the mammary gland [201].

#### 3.1.3. The Autoinflammatory Signaling Loop Mediated by AhR and KYN/KYNA

Trp is a source of endogenous ligands of AhR, such as FICZ and 2-(1′H-indole-3′-carbonyl)-thiazole-4-carboxylic acid methyl ester [ITE] [11], as well as of metabolites of the KP: KYN and KYNA [81,84,85,86,141].

KYNA, compared to KYN, is a more potent and stabler ligand of AhR [58] and, just as KYN, promotes the production of inflammatory cytokines [81]. AhR positively regulates the expression of IDO and, consequently, the production of KYN in immune cells [205]. KYN and KYNA, agonists of AhR, take part in a positive feedback loop in AhR signaling [206]; this loop may control an inflammatory response via AhR.

First of all, activated AhR induces the expression of proinflammatory IL-6 [207,208]. Second, IDO1 maintains its own expression through the autocrine AhR–IL-6–STAT3 signaling loop [93,207] because inhibition of the expression of IL-6 or of STAT3 has been shown to reduce the expression of IDO1 [207,209]. The IL-6–IDO1–AhR loop contributes to the accumulation of KYN and KYNA by accelerating the metabolism of the Trp–KYN–KYNA axis. In dendritic cells, ligands of Toll-like receptors, e.g., LPS, trigger transcriptional activation of STAT-1 and NF-κB, thereby inducing the expression of IDO and subsequent synthesis of KYN, which activates AhR [206]. Third, in inflammatory conditions, induction of AhR is possible. Such data were obtained in a study on the activity of AhR in human and mouse CD4-positive T cells under Th17 polarization conditions after simultaneous administration of IL-6 and transforming growth factor beta (TGFβ); the study revealed that AhR is transcriptionally regulated by IL-6 [186].

Confirmation of the finding that AhR is a downstream effector of IL-6 was obtained in a study on HepG2 cells after treatment with a multifunctional cytokine belonging to the IL-6 family: oncostatin; the results showed enhancement of the transcription of the *AhR* gene and expression of the AhR protein [210].

STAT3, which is a major effector of the IL-6 cytokine family, has also been found to bind to a STAT motif in the *AhR* promoter, thereby enhancing transcription of the *AhR* gene and biosynthesis of the AhR protein. It has been hypothesized that the IL-6 cytokine family modulates AhR expression through STAT3 activation [210]. Finally, a possible mechanism controlling the inflammatory response involving AhR is IDO1 phosphorylation by an AhR chaperone: pp60src [211].

**Figure 3:**
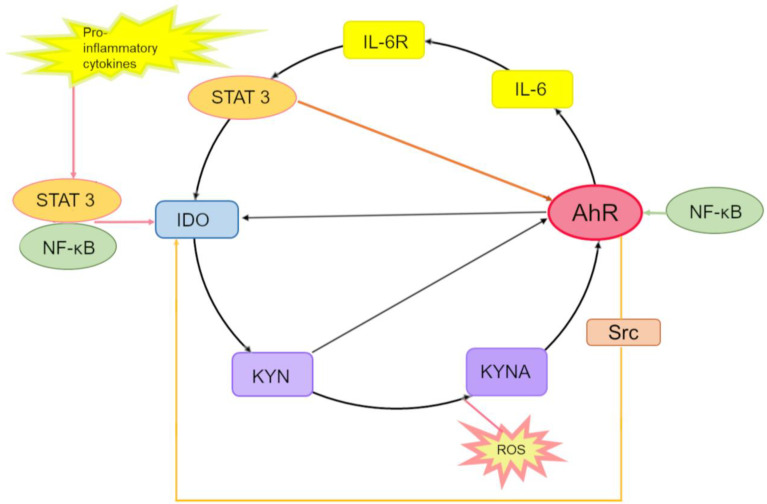
The interleukin (IL)-6-, signal transducer and activator of transcription (STAT)3-, IDO-, KYN-, KYNA- and AhR-driven autoinflammatory loop. In the cytoplasm, proinflammatory cytokines induce the expression of the IDO-1 enzyme through activation of STAT transcription factors and NF-κB. Kynurenic acid (KYNA) arises in the IDO-dependent canonical pathway or alternatively via direct transformation of either KYN or Trp by reactive oxygen species (ROS). KYN and KYNA activate AhR, which binds to the XRE in the promoter of IL6 and participates in the production of IL-6, thereby enhancing inflammation. By binding to its receptor IL-6R, IL-6 promotes the activation of STAT-3, which binds to promoters of IDO-1 and AhR, thus inducing the expression of IDO-1 and AhR genes. Activation of AhR (by KYN)—which, just as STAT-3, binds to the IDO1 promoter—contributes to IDO-1 expression initiation. Enzymatic activation of IDO-1 is also mediated by pp60src, which dissociates from an inactivating AhR complex and phosphorylates IDO-1, thereby completing the inflammatory cycle. Interaction of the KYNA–AhR complex with NF-κB may also take part in the induction of IL6. Besides, ligand-activated AhR initiates the activation of the protein tyrosine kinase Src and, thereby, IDO-1 phosphorylation.

In summary, we can conclude that IL-6, STAT3, IDO, KYN, KYNA, and AhR may form an AhR-driven autoinflammatory loop that consists of the following steps (Figure 3).

In the cytoplasm, IDO1 catalyzes the formation of KYN from Trp, then KYN is converted into KYNA, and both can activate AhR. KYNA is produced either by an IDO-dependent canonical pathway (via direct transformation of Trp or KYN) or by an alternative pathway under the action of reactive oxygen species.

Activated AhR binds to an XRE in the *IL6* promoter and thus participates in the endogenous production of IL-6 and in the enhancement of the inflammatory state. By binding to its receptor IL-6R, IL-6 leads to the activation of STAT3, which binds to promoters of *IDO1* and *AhR*, thereby inducing the expression of these two genes.

Activated AhR associates with the *IDO1* promoter and activates the expression of IDO1. Enzymatic activation of IDO1 is also accomplished by pp60src, which dissociates from the AhR-inactivating complex and phosphorylates IDO1, thus completing this inflammatory cycle.

In parallel, STAT3 maintains the expression of AhR, thereby stimulating the expression of IL-6 and hence creating a self-perpetuating autoinflammatory loop.

Furthermore, activation of STAT3 can stimulate the noncanonical NF-κB pathway and lead to induction of *IDO* transcription through direct binding to a promoter region of *IDO* in dendritic cells and myeloid-derived suppressor cells [212,213]; besides, the noncanonical AhR signaling pathway can be involved in NF-κB–mediated inflammation [171,172,173,199].

## 4. The Role of KYNA in Inflammation in Various Pathologies

Regarding chronic inflammatory conditions, it is believed that chronic stress or low-grade inflammation can induce the production of KYNA [58]. In many cases, however, it remains unclear whether the upregulation of KYNA is a primary disorder or a compensatory response due to inflammatory signal transduction [58].

An altered immune response in schizophrenia, including neuroinflammation and an imbalance of Th1 and Th2 branches [214,215,216], has been reported to be connected with a Trp/KYN metabolic imbalance, leading to enhanced production of KYNA in the brain and, therefore to an imbalance of glutamatergic neurotransmission [217]. Immunological and neurochemical imbalances cause a chronic proinflammatory state in combination with higher production of PGE2 and proinflammatory cytokines, cyclooxygenase-2 expression, and hypofunction of the NMDA receptor [215,217].

KYNs, including KYNA, have been linked to inflammatory pathways taking part in inflammatory bowel diseases. In such patients, IDO is overexpressed, the amount of Trp is reduced, and the KYN/Trp and KYNA/Trp ratios are elevated [79]. Pathogenic *Escherichia coli*, a common etiological factor of ulcerative colitis, is capable of converting KYN into KYNA by means of aspartate aminotransferase [218,219]. It has been demonstrated that the level of KYNA in ulcerative colitis and Crohn’s disease is higher during periods of intense inflammation than in periods of remission [220], whereas the ratio of KYNA to Trp positively correlates with stronger inflammation, greater histological damage, and the number of surgical operations and of hospitalizations in patients with ulcerative colitis [221].

Type 2 diabetes mellitus is one of the risk factors for CVDs. In turn, chronic stress and low-grade chronic inflammation are important risk factors for type 2 diabetes mellitus [222,223,224]. Stress hormones (chronic stress) and/or inflammatory factors, including low-grade chronic inflammation, activate enzymes in the first stages of the KP [225,226]. Pyridoxal 5-phosphate (P5P) is required as a cofactor for the KAT that converts KYN into KYNA and for kynureninase, which transforms 3-hydroxykynurenine into 3-hydroxyanthranilic acid. During inflammation, the need for P5P is greater, and its deficiency may develop. Nonetheless, kynureninase is more sensitive to P5P deficiency than KYN aminotransferase is, and hence, metabolism deviates toward the biosynthesis of KYNA [226,227,228].

Levels of KYNA, along with KYN and xanthurenic acid, in plasma samples are reported to be higher in patients with type 2 diabetes mellitus than in nondiabetic patients [226]. Although xanthurenic acid is considered diabetogenic [229,230], KYNA, according to some data, can enhance glucose secretion in the liver owing to the antagonism of NMDA receptors in the dorsal nucleus of the vagus nerve [231] and, according to other data, can inhibit proinsulin synthesis in rat pancreatic islets of Langerhans [232].

Other evidence suggests that KYNA has antidiabetic effects by reducing inflammation and insulin resistance in adipocytes and muscle cells via stimulation of the receptor GPR35 [75,233]. Additionally, Goto-Kakizaki rats (a nonobese model of type 2 diabetes mellitus) exhibit lower plasma levels of KYNA as compared to the wild type; oral administration of KYNA delays the development of diabetes in Goto-Kakizaki rats and stimulates the expression of uncoupling protein (UCP) in HepG2 cells and mouse hepatocytes at mRNA and protein levels [234].

The KP, including KYNA, is implicated in obesity, which is a risk factor for CVDs. Expression of enzymes of the KP, including KAT III, is elevated in the omental adipose tissue of obese women and is induced by proinflammatory cytokines in primary human adipocytes [235]. Elevated levels of KYNA in a woman’s plasma during early pregnancy (18 weeks of gestation) correlate with a significantly higher risk of preeclampsia, i.e., pregnancy-specific hypertension, in obese women [236].

KYNA may participate in various metabolic disorders and act as a regulator of inflammation and energy homeostasis by enhancing energy metabolism in adipose tissue [234,237,238]. KYNA increases energy utilization by activating GPR35, which stimulates lipid metabolism and the expression of thermogenic and anti-inflammatory genes in adipose tissue [237].

In C2C12 and 3T3-L1 cells, as well as in skeletal muscle and subcutaneous adipose tissue of mice, it has been found that KYNA can alleviate palmitate-induced inflammation and insulin resistance by means of pathways mediated by GPR35/AMPK and SIRT6 [239]. Presumably, the influence of KYNA on adipocytes depends on such biomolecules as PGC-1α and PRDM16, which are important for the formation of adipose tissue [237,240,241].

KYNA serves as a modulator of immune cells located in adipose tissue [237]. Mice fed a high-fat diet and given daily KYNA gain less weight and have better glucose tolerance; however, in mice with a genetic *Gpr35* deletion, these effects of KYNA are absent, suggesting that this function of KYNA is mediated by signaling through GPR35 [237]. An increase of the kynurenic acid level to “exercised” levels is sufficient to produce an anti-inflammatory phenotype in adipose tissue: overexpression of anti-inflammatory cytokines and underexpression of inflammatory markers such as TNF [237].

After a risk assessment of progression to end-stage renal disease in patients with type 2 diabetes mellitus, high levels of Trp, KYNA, and xanthurenic acid proved to be associated with a slower decline of the estimated glomerular filtration rate, and the opposite was true for the KYN/Trp ratio [242]. A protective function of KYNA toward kidneys has been documented in animal studies [243,244], including rats with spontaneous hypertension [244].

The production of kynurenic and xanthurenic acids is mediated by a KAT whose underexpression in carotid artery plaques is associated with increased inflammation and a higher risk of cerebrovascular events [245]. Of note, the expression of KAT is under the control of peroxisome proliferator-activated receptor coactivator 1-alpha and correlates with the mitochondrial content [246], and mitochondrial dysfunction is a hallmark of progressive diabetic kidney disease [242].

## 5. KYNA in the Cardiovascular System

Inflammation and immune activation play important roles in the pathogenesis of CVDs [247,248,249,250,251]. Interactions of various cells of the adaptive and innate immune systems with inflammatory mediators modulate acute and chronic inflammation, causing various diseases. The coordination of inflammatory mechanisms triggers extracellular-matrix remodeling, oxidative stress, tissue damage, angiogenesis, and fibrosis in various tissues. Inflammatory mechanisms participate in most CVDs and complications, including hypertension, coronary heart disease, myocarditis and atherosclerosis [252].

Metabolites of the KP play an important part in the physiology and pathology of the cardiovascular system by influencing vascular inflammation [253] and by taking part in the initiation and progression of CVDs [8,51,254,255], including myocardial infarction, unstable angina pectoris, and sudden cardiac death [256,257].

Metabolites of the KP have been described as potential diagnostic and prognostic biomarkers of CVDs [51]. In particular, it has been shown that the KYN/Trp ratio may be positively associated with progressive atherosclerosis and coronary heart disease [255,258].

Proinflammatory signals promote Trp degradation through the KP, thereby leading to the formation of several immunomodulatory metabolites [255]. In the cardiovascular system, endothelial cells produce large amounts of KYNs, especially KYNA [259]. It is possible that events causing endothelial dysfunction may impair the KP, and changes in the amounts of metabolites produced by the KP may, in turn, modulate an inflammatory process [255].

### 5.1. KYNA in Heart Disease

KYNA is reported to be associated with such pathological conditions as myocardial infarction, cardiac arrest, heart failure, post-stroke changes, myocarditis, hypertension, and atherosclerosis. In some studies, the upregulation of KYNA relative to other metabolites of the KP is regarded as an adverse factor in patients with CVDs.

Hyperactivation of the KP and elevated plasma levels of KYNA, hydroxykynurenine, anthranilic acid, and hydroxyanthranilic acid in patients with stable angina pectoris are associated with a high risk of acute myocardial infarction [8,258].

Higher plasma levels of these metabolites are observed in patients having cardiac arrest with an initial nonshockable rhythm (than in such patients with a shockable rhythm), in patients with lower 24 h systolic blood pressure (systolic BP), and in patients who died in an intensive care unit (than in those who survived) [8,260].

Heart failure correlates with elevated plasma levels of KYNA, KYN, and quinolinic acid [261,262]. In patients with heart failure, levels of inflammatory cytokines IL1β and TNF are increased; that is, chronic and systemic inflammation may be a trigger of this disorder [263,264], and these cytokines mediate activation of the KP [95,103,104]. KYNA, anthranilic acid, 3-hydroxykynurenine, and 3-hydroxyanthranilic acid worsen myocardial mitochondrial respiration, thereby causing disturbances of energy and calcium homeostasis in the heart and greater production of reactive oxygen species and possibly contributing to the development of heart failure [265,266].

On the other hand, KYNA exerted a cardioprotective effect in a study on rodents [267]. KYNA has been identified as a key metabolite whose level is altered after acute kidney injury, and that ensures cardiac protection after ischemia. KYNA in drinking water of animals that had a myocardial infarction improved their heart recovery [267]. It was found in vitro that the protection of cardiomyocytes is mediated by a mechanism partially involving mitochondrial protection [267].

In experiments on rat myocardial H9C2 cells and on rats, they were subjected to hypoxia/reoxygenation or myocardial infarction in the presence or absence of KYNA [268]. The addition of KYNA significantly reduced the death of H9C2 cells and the magnitude of myocardial damage in the rats. Under the influence of KYNA, *SOD2* mRNA levels increased, whereas ERK1/2, AKT, and FOXO3α phosphorylation levels decreased. Overall, KYNA significantly diminished the risk of myocardial ischemia/reperfusion injury in both in vitro and in vivo models. Thus, KYNA-mediated cardioprotection is related to enhanced mitophagy and antioxidant defense [268].

In KMO^−/−^ mice, there are elevated serum levels of KYN, anthranilic acid, and KYNA and significantly lower levels of some chemokines; therefore, it has been hypothesized that manipulation of the functioning of KP enzymes may help improve the outcome of myocarditis [51,269].

### 5.2. KYNA in Vascular Diseases

More active IDO1 and higher concentrations of several metabolites of the KP, including KYNA, are reported to correlate with higher mortality and post-stroke cognitive impairments in patients [270,271]. Different (opposing) effects of various metabolites of the KP have also been demonstrated in animal models of stroke [272,273,274], but a KYNA analog has manifested neuroprotective properties in transient forebrain ischemia in a rat model [275].

KYNA promotes the restoration of rat aortic functionality after ischemia followed by reperfusion, and this phenomenon may be due to the antioxidant activity of KYNA and the prevention of endothelial damage during reperfusion [276]. Animal models also suggest that levels of KYNA and 3-hydroxyanthranilic acid go up in plasma after cardiopulmonary resuscitation applied to cardiac arrest in rats and pigs [277].

KYNA can selectively reduce heart rate in rats without appreciably affecting BP [36].

Endogenous KYNA in the brain likely participates in BP control. KYNA concentration and KAT activity are lowered in the brain of spontaneously hypertensive rats (SHR rats) compared to controls (Wistar–Kyoto rats) [278]. In SHR rats, the activity of the sympathetic nervous system is higher than normal, and stimulation of BP control centers by glutamate and nicotine raises BP. KYNA, which is an antagonist of glutamate and nicotinic acid, is synthesized in the brain by KAT I, which in SHR rats carries missense mutation E61G; this observation may explain the increased sensitivity of SHR rats to glutamate and nicotine and the predisposition of these rats to hypertension [279].

Elevated serum concentrations of the sulfur-containing amino acid homocysteine are known to be associated with the development of atherosclerotic lesions and have a detrimental effect on blood vessels by increasing the risk of coronary heart disease and stroke. KYNA has been found to counteract the harmful effects of homocysteine on endothelial cells in vitro [280]. It has been shown that the elevated levels of KYNA observed in patients with hyperhomocysteinemia apparently protect them from homocysteine-mediated atherosclerosis [281]. On the other hand, aortic stiffness positively correlates with serum homocysteine levels and with KYNA levels in patients having atrial fibrillation, either hypertensive or normotensive [282,283].

High concentrations of KYNA in atheromatous plaques are associated with plaque instability in human atherosclerotic lesions, whereas in stable plaques, KYNA either is not detectable or is present in small amounts [8,284].

## 6. AhR in the Cardiovascular System

Although the AhR expression level in the heart and blood vessels is not very high, AhR is closely linked with the normal development of the heart and blood vessels [32,285] and with the progression of CVDs [32,286,287].

During embryonic development, cardiogenesis is sensitive to AhR expression [286], indicating the vital importance of AhR signaling for cardiac development [286] and the decisive role of AhR in cardiomyocyte differentiation [32]. Apparently, AhR activation during the differentiation of embryonic stem cells disturbs the expression of genes of TGFβ–BMP2/4 and WNT signaling pathways (which are crucial for the ontogenesis of the cardiac mesoderm), thereby causing a loss of contractility in the resulting lineage of cardiomyocytes [288]. It has been shown that in mouse embryonic stem cells, AhR must be repressed for the regulation of mitotic progression and pluripotency. In cells that have escaped AhR repression, levels of pluripotency factors OCT4 and SOX2 are lowered, and the duration of mitosis is increased due to an impairment of the MID1–PP2A–CDC25B–CDK1 signaling cascade. During premature loss of pluripotency owing to AhR expression, cardiomyocyte progenitor cells differentiate into neuroglial cells [289].

AhR may be implicated in the pathogenesis of cardiac hypertrophy. For instance, it is reported that in mice with an *AhR* knockout, hypertrophic changes in the heart develop, as does cardiomyopathy with decreased cardiac output, possibly owing to cardiomyocyte hypertrophy [21] or to elevated blood pressure and increased plasma concentrations of endothelin 1 (ET-1) and angiotensin II [290]. The diminished cardiac output and lower aortic pressure in AhR-deficient mice may be due to disturbances of the renin–angiotensin system and activation of ET-1 signaling [32,291].

AhR participates in the regulation of physiological functions of blood vessels, including vascular development as well as angiogenesis. AhR deficiency, just as abnormal activation of AhR, can lead to vascular pathology [32]. In AhR^−/−^ mice, there are aberrations of angiogenesis: abnormal vascular structures in the liver and kidneys [285].

It is known that AhR can prevent the interaction of ARNT with HIF-1α and can suppress the expression of vascular endothelial growth factor (VEGF), thereby ultimately blocking angiogenesis [32]. For instance, during AhR deficiency, ischemia-induced angiogenesis is more active with the participation of the HIF-1α–ARNT complex and of a HIF-1α target gene: *VEGF*. Because ARNT is a partner of both AhR and HIF-1α, it is likely that AhR competes with HIF-1α for ARNT, thus limiting VEGF production [292].

### 6.1. The Role of AhR in Cardiovascular Pathologies

AhR deficiency, just as abnormal activation of AhR, leads to vascular dysfunction and many vascular diseases. Activation of AhR by its agonists correlates with an elevated risk of cardiovascular problems [26,293]. This notion is supported by some epidemiological studies and animal experiments [27,28,29].

AhR is a major participant in the pathogenesis of such cardiovascular pathologies as myocarditis, hypertension, coronary heart disease, and pulmonary arterial hypertension. AhR-related mechanisms of these diseases include inflammatory and immune responses, oxidative stress, and endothelial dysfunction [32].

In myocarditis, the involvement of AhR is mainly due to its participation in immune system functioning [32]. The main etiology of myocarditis is an infection, although the etiopathogenesis of this disease is closely related to immune and inflammatory reactions [294]. AhR takes part in the regulation of innate and adaptive immune responses to infection, whereas AhR deficiency exacerbates inflammation caused by some microorganisms [32]. AhR plays an important role in the pathogenesis of the myocarditis that develops during *Trypanosoma cruzi* infection: in infected mice, parasitemia and the inflammation and fibrosis of the myocardium are significantly weaker in the AhR^−/−^ group than in the wild type group owing to lower levels of reactive oxygen species and of some cytokines [295].

Effects of classic AhR ligands on the body may increase the risk of hypertension [27] and atherosclerosis [296]. It has been demonstrated that 3-methylcholanthrene can induce hypertension mediated by inactivation of endothelial NO synthase (eNOS) in mice [297], whereas in AhR^−/−^ mice, hypotension is observed [298]. It has been found that polymorphisms of genes related to the AhR signaling cascade are closely related to the pathogenesis of essential hypertension [299].

There are some data indicating that exposure to exogenous ligands of AhR (either dioxins or polycyclic aromatic hydrocarbons) raises the risk of coronary heart disease [300]. Circulating AhR is upregulated in patients with coronary heart disease, and polymorphisms of the *AhR* gene may mediate the risk of this disease [301]. A higher risk of coronary heart disease may result from an interaction of AhR with transcription factor TCF21; the latter has been shown to play a part in changes in the phenotype of smooth muscle cells in response to vascular damage [302]. Besides, after myocardial ischemic injury, AhR stimulates the expression of inflammatory cytokines, including IL-1β and IL-6, and of C-reactive protein (CRP) [303].

Involvement of AhR in acute ischemic stroke has been demonstrated by pharmacological and genetic approaches to AhR loss of function in a mouse model of middle cerebral artery occlusion, where ischemic injury upregulates the AhR protein and its transcriptional activity in neurons [304]. In this context, it was found that in the mouse model of the brain after stroke, the activation of AhR is mediated by its endogenous ligand KYN [304].

Participation of AhR in atherosclerosis is indicated by evidence that exposure to contaminants containing AhR ligands (either dioxins or polycyclic aromatic hydrocarbons) contributes to the initiation and progression of this disease [305,306,307].

### 6.2. AhR and Inflammation in CVDs

CVDs have a multifactorial etiology. An important role in the initiation and progression of CVDs is played by low-grade persistent inflammation characterized by upregulation of circulating proinflammatory cytokines [such as TNF and interleukins (IL-1β and IL-6)] and of circulating CRP, which are independent risk factors of mortality and morbidity of such diseases [255]. Vascular inflammation also includes activation of nucleotide oligomerization domain-like receptor family pyrin domain containing 3 (NLRP3) inflammasomes and *MMP*s [308,309]. Accumulating data suggest that inflammatory processes in vascular and cardiac tissues are linked with the onset of such pathological conditions as myocarditis, coronary heart disease, hypertension, and atherosclerosis [252,309].

Coronary heart disease is a group of pathologically related conditions characterized by atherosclerosis of cardiac arteries and potential functional impairment of the coronary circulation [310].

Atherosclerosis affecting large and medium-sized arteries is the leading trigger and cause of most cardiovascular morbidity and mortality worldwide [2,3]. Atherosclerosis is now generally recognized as a chronic inflammatory disease with autoimmune factors [311]. Atherosclerosis pathogenesis involves immune inflammatory cells, mainly macrophages, T cells, B cells, dendritic cells, and mast cells [312].

Inflammation plays a crucial part in all stages of atherosclerotic plaque formation. During the initiation of atherogenic processes, activation of endothelial cells by oxidized lipoproteins leads to the expression and release of numerous inflammatory cytokines (such as IL-1β and TNF), a chemoattractant, and adhesion molecules [312], resulting in the infiltration of arterial walls by leukocytes and monocytes and eventually in inflammation [313,314]. Inflammation also participates in the late stage of atherosclerosis by enhancing local accumulation of macrophages, which weaken the fibrous lining of atherogenic plaques by secreting collagen-degrading *MMP*s [315].

The aforementioned plaque-resident cells—macrophages, dendritic cells, T cells, B cells, hematopoietic stem cells, and foam cells—all overexpress AhR [311]. AhR has been shown to contribute to inflammation regulation in atherosclerosis. This phenomenon may be based on enhanced chemotaxis of monocytes (which are targets for polycyclic aromatic hydrocarbons); this chemotaxis may be due to the signaling of inflammatory factors (e.g., VCAM-1) via the AhR–NF-κB pathway [316,317]. Additionally, AhR may be implicated in the absorption of oxidized low-density lipoproteins by macrophages, which gives rise to foam cells.

AhR activity is regulated and triggered by endogenous ligands, which include oxidized low-density lipoproteins and Trp metabolites, which accumulate in atherosclerotic plaques and affect atherosclerosis progression [245]. In foam cells, AhR signaling induces the expression of proinflammatory interleukins IL-1β, IL-8, and TNF, thereby promoting atherosclerosis development [22]. The participation of AhR in foam cell formation is supported by an in vitro work suggesting that the inflammation mediating cholesterol accumulation in foam cells is caused by particulate matter and is an early sign of CVDs [191,318].

Accelerated proliferation of vascular smooth muscle cells is a critical factor in the development of vascular complications [319]. It is possible that the proliferation of vascular smooth muscle cells is induced by an AhR-driven launch of the NF-κB signaling pathway and the production of reactive oxygen species, as demonstrated, for example, during activation by an AhR ligand called indoxyl sulfate [319].

Mechanisms by which AhR can suppress inflammation may include the induction of anti-inflammatory Treg cells and the downregulation of proinflammatory cytokines. Many studies indicate that AhR activation by a ligand can increase the number of Treg cells while attenuating inflammation and improving the course of inflammatory disease [320,321]. AhR activation may participate in the prevention of excessive induction of proinflammatory cytokines in response to an inflammatory stimulus in various cells, including fibroblasts, endothelial cells, and macrophages [23,24,25]. The ability of the AhR to downregulate proinflammatory cytokines is an important mechanism, but this effect depends on an AhR-activating ligand and on the cell type involved [320]. Both canonical and noncanonical AhR signaling pathways can regulate inflammatory processes via the expression of IL-10, IL-21, and IL-22 and through the differentiation and expansion of Treg cells (such as CD4^+^ cells, resident memory T cells [T_RM_], and T_H_17 cells) [18,322,323,324,325].

## 7. Interaction between the AhR Pathway and Trp Metabolism in the Initiation of the Pathophysiological Processes and Diseases Associated with the Cardiovascular System

As mentioned above, enhanced activation of the KP is associated with risk factors and increased risk of CVDs, according to conclusions of studies based on comparisons of patients with CVD and healthy controls [245]. Cells of the heart and vascular system express AhR-regulated genes, many of which take part in the pathogenesis of CVDs [326]. KP metabolites and AhR are involved in inflammatory processes. Inflammation is reported to be modulated by several metabolites of the KP [327], for example, by KYNA, which inhibits the secretion of IL-6, TNF, and IFNγ by immune cells [82,90,328]. The interaction between AhR and KYN promotes the formation of Treg cells [34]. A recent paper indicates that IL4I1 (secreted L-amino acid oxidase) launches the AhR pathway through the production of KYNA [329]; IL4I1 is expressed in macrophages, mature dendritic cells, T cells, and B cells stimulated by IL-4.

Data are accumulating regarding the participation of KP metabolites and AhR signaling in inflammatory processes in vascular and cardiac tissues associated with the initiation of such pathological conditions as coronary heart disease, myocardial infarction, and atherosclerosis [8,245,252,258,267,309].

Above, we described the general understanding of possible interactions of KYN, KYNA, and AhR according to experimental studies. New knowledge about the interaction of KYNA with AhR in inflammatory diseases of the heart and blood vessels is presented in the present section.

Trp metabolism and the AhR signaling pathway can be altered by inflammation or interact in an interdependent manner, thereby leading to inflammation and the pathophysiological processes and diseases related to the cardiovascular system. We are still far from understanding the exact triggers and molecular pathways of such events. At present, there are few research articles proving the importance of the interaction of KP metabolites with the AhR signaling cascade for various outcomes of CVDs.

It has been found that the AhR activated by metabolites of the KP takes part in such an inflammatory disorder (key for CVDs) as atherosclerosis [330]. KP disturbances linked with weaker regulation of enzymes of the KYNA branch of the pathway are associated with unstable atherosclerosis [245,258]. A recent study uncovered a significant reduction in the activity of the KYNA branch (this phenomenon has never been reported in the context of atherosclerosis) and revealed that an important mechanism in the regulation of vascular inflammation is KYNA-mediated signaling through AhR [245].

In that work, investigators characterized the expression profile and activity of the key enzymes of two main branches of the KP in human atherosclerotic diseases depending on the severity of carotid atherosclerosis [245]. Their findings indicated reciprocal regulation between inflammation and the two major branches of this pathway in the vascular wall: the quinoline branch and the KYNA branch. Transcriptomic analysis of carotid atherosclerotic plaques and of control arteries suggested that the expression of quinoline branch enzymes is elevated in carotid plaques, whereas the expression of KAT1 and KAT2 from the KYNA branch is low [245].

It has been demonstrated that IFNγ or a combination of TNF with IL-1β can suppress the expression of a KAT in various cells, including macrophages and dendritic cells [63,331]. It is likely that IFNγ, TNF, and IL-1β may influence KAT expression in immune and nonimmune cells because numerous cytokines are present in plaques [332,333]. Analysis of expression correlations between enzymes of the KP and genes related to inflammation and plaque stability has shown dichotomous roles of the two branches of the KP, where the quinoline branch is proinflammatory, whereas the KYNA branch protects from inflammation [245].

Thus, the characterization of the KP in human carotid plaques indicates that unstable atherosclerotic disease is associated with impaired production of KYNA in plaques. Moreover, this disturbance of the KP has been linked with the inflammatory markers (CCL5 and CXCL10) that are typical of activated macrophages. Consequently, diminished activity of the KYNA branch is associated with stronger inflammation and decreased plaque stability [245].

The same work showed in vitro and in vivo that KYNA transmits signals through AhR by inhibiting the activation of macrophages and the migration of leukocytes; in other words, KYNA-mediated signal transduction through AhR is the main regulator of human-macrophage activation and of the recruitment of proinflammatory cells, whereas KYNA signaling via AhR is the main mechanism underlying downregulation of *CCL5* and *CXCL10*, which are target genes of AhR [245]. Overall, these data indicate that KYNA signaling through AhR is a protective mechanism in the arterial wall.

It is worth mentioning that some evidence has been reported regarding the detrimental effect of KYNA on atherosclerosis development. These data have been obtained in an atherosclerosis model based on mice deficient in IDO [284]. It was found there that administration of physiological amounts of KYNA to the mice deficient in IDO limits the expression of the major immunoregulatory atheroprotective cytokine IL-10 and accelerates atherosclerosis progression. It was demonstrated that IDO activity is necessary for IL-10 expression regulation via the production of downstream KYNA [284].

One can hypothesize that AhR activation would abrogate the adverse role of KYNA in atherosclerosis development in IDO-deficient mice because it is known, for example, that AhR promotes IL-10 expression in inflammatory macrophages via the nongenomic Src–STAT3 signaling pathway [334].

The fact that KYNA-mediated effects may differ among cell types and stimulation conditions [82,90,91,109] requires further research into the functions of this metabolite in responses of immune and vascular cells within the arterial wall.

In a study on the relation of AhR signaling with the risk and complications of diseases associated with atherosclerosis, results revealed a negative role of AhR in atherosclerosis [302]. It was shown there that AhR is overexpressed in vascular smooth muscle cells (VSMCs) of carotid artery plaques compared to control arteries. A mechanism of this upregulation was proposed: possible interaction of transcription factor 21 (TCF21) with AhR during activation of inflammatory genes in VSMCs [302].

There is also evidence suggesting that AhR can have bidirectional effects on inflammation regulation depending on the availability of exogenous ligands of AhR. For instance, in the absence of exogenous ligands, AhR can mediate proinflammatory effects and promote atherosclerosis progression [335].

In this regard, one should realize that the molecular events connected with the participation of KP metabolites and AhR signaling in the development of atherosclerosis and its outcome depend on a cellular context.

Atherosclerotic plaque rupture is the main cause of acute myocardial infarction and stroke [336]. Arterial calcification is now recognized as an active osteogenic process reminiscent of osteoblast differentiation during skeletal mineralization, and the major contributors are VSMCs [337]. The formation of atherosclerotic plaques in vascular atherosclerotic lesions is promoted by increasing arterial stiffness caused by the calcification. A recent paper uncovered the crucial involvement of Trp metabolism in the modulation of VSMC calcification, along with a key role of IDO1 [338]. This was the first study to show that IDO1 reduces arterial calcification through KYN production in VSMCs but not in macrophages. IDO1 underexpression exacerbates the progression of osteogenic reprogramming of VSMCs and the subsequent calcification [338].

Calcification has been prevented by the administration of KYN both in vitro and in vivo and has proven to be dependent on Runt-related transcription factor 2 (RUNX2), which is necessary and sufficient for osteogenic differentiation and calcification in blood vessels [339]. RUNX2 is known to undergo many post-translational modifications, including phosphorylation, acetylation, and ubiquitination [339]. It has been demonstrated that KYN stimulates RUNX2 degradation, whereas RUNX2 stability, which is regulated by KYN, is mediated by AhR. AhR acts in a noncanonical manner as an atypical component of the E3 ubiquitin ligase complex containing Cul4B, thereby facilitating ubiquitin-mediated proteasomal degradation of RUNX2 with consequent RUNX2 suppression [338].

Thus, the AhR activated by KYN (a key metabolite of Trp in the KP) mediates RUNX2 degradation by interacting with the E3 ubiquitin ligase via the noncanonical pathway; this effect may represent a new approach to calcification reduction in vascular diseases. This first paper in this field highlights the importance of Trp metabolism and KYN in arterial calcification and in atherosclerosis and provides a rationale for further investigation of KP metabolites, especially KYNA, as protective molecules in this disease.

Myocardial infarction, as a type of ischemic CVD, drives left-ventricle adverse remodeling by causing damage to cardiomyocytes and vascular cells. The cellular alterations in myocardial infarction involve a wide range of molecular pathways, including KYN metabolism and the IDO1–KYN–AhR axis [340].

The concentration of circulating AhR can influence the susceptibility to (and progression of) coronary heart disease [301], and AhR takes part in myocardial ischemia-reperfusion injury by modulating mitochondrial apoptosis [341]. In a model of myocardial ischemia based on the occluded left anterior descending artery, it has been found that acute myocardial ischemia can result in substantial expression of AhR in the necrotic myocardium, activate AhR, and induce inflammation [32]. Additionally, animal studies have revealed a decrease in AhR expression following the improvement of cardiac health after ischemic injury [342].

It is reported that some AhR ligands, such as BaiCalin1, can reduce myocardial necrosis and inflammation by inhibiting cardiac AhR expression [303,341]. The AhR pathway is cardioprotective against cardiotoxicity and induces heart-specific transcriptional responses [343,344].

Regarding KYN biosynthesis with the help of IDO, KYN is markedly upregulated after myocardial infarction, and a genetic deletion or pharmacological inhibition of IDO attenuates cardiac damage and cardiac dysfunction after myocardial infarction [345]. After myocardial infarction, KYN induces cardiomyocyte apoptosis through the production of reactive oxygen species via an AhR-dependent mechanism [345].

Currently, there are few experimental studies aimed at deeper elucidation of the cellular and molecular mechanisms behind the interaction of KP metabolites with AhR in cardiac tissues affected by myocardial infarction. Such research has been performed on Wistar rats that have the occluded left anterior descending artery and are subjected to one of two exercise protocols: moderate-intensity continuous training or high-intensity interval training [346]. In that paper, it was concluded that myocardial infarction disturbs the balance of the IDO1–KYN–AhR axis in cardiac cells and triggers oxidative stress. Both training protocols significantly lowered the KYN level in the heart tissue of the rats with myocardial infarction, but the effect of high-intensity interval training was greater than that of moderate-intensity continuous training [346].

Other investigators have analyzed cardiac functional recovery in mice deficient in IDO within specific endothelial or smooth muscle cells, cardiomyocytes, or myeloid cells after acute myocardial infarction [345]. Their study presents complex crosstalk between cardiac endothelial cells and cardiomyocytes during cardiac recovery after myocardial infarction and implies adverse participation of endothelial IDO (in cardiac repair) through KYN production [345].

KYN biosynthesis with the help of IDO is reported to be markedly induced after myocardial infarction in mice [345]. In that article, complete genetic deletion or pharmacological inhibition of IDO attenuated cardiac damage and cardiac dysfunction after myocardial infarction. In this context, IDO loss of function in smooth muscle cells, inflammatory cells, or cardiomyocytes did not affect cardiac function and remodeling in the mice that suffered the heart attack. By contrast, an endothelial-cell–specific knockout of *IDO* improved cardiac function and cardiomyocyte contractility and diminished ventricular adverse remodeling. Administration of KYN to the IDO-deficient mice in vivo abrogated the protective effects of the *IDO* knockout [345].

It was also found there that the apoptotic effects on cardiomyocytes—as well as KYN-mediated induction of reactive oxygen species production—were dependent on AhR because its inactivation prevented the KYN-mediated effects [345]. Overall, these data suggest that IDO can be a crucial player in local regulation of cardiac homeostasis after myocardial infarction and underscore paracrine effects of KYN-caused apoptosis on myocardial infarction mediated by endothelial cells.

## 8. Conclusions

Substantial research advances have been made in the field of CVD biomarkers, but it is estimated that there is a need for new, more sensitive and CVD-specific biomarkers in clinical practice for the identification of high-risk groups in terms of CVDs and for accurate diagnosis and theranostics within the framework of precision personalized medicine [347,348,349,350].

In several studies, KYNA is considered a potential biomarker of medical conditions associated with CVDs. For instance, the ratio of quinolinic acid to KYNA has been proposed as a biomarker of post-stroke cognitive decline [351]. Furthermore, KYNA—among other metabolites of the KP—is being investigated as a possible biomarker of coronary heart disease [255].

The present review provides up-to-date information on important biological functions of KYNA and is focused on its anti-inflammatory properties and its connection with the AhR signaling pathway.

The onset of many pathophysiological processes and diseases caused by inflammation is related to the AhR pathway and to Trp metabolism along the KP. In recent years, links of KYNA with the immune system, inflammation, and the cardiovascular system have become more obvious. Because of these connections, the anti-inflammatory functions of KYNA are especially interesting in the context of evidence that the KP is a compensatory mechanism that is launched in some CVDs and carries out a protective function. Due to the proven anti-inflammatory significance of KYNA, it can be considered a target and agent for therapeutic interventions in several diseases. The link between KYNA upregulation in the body and regular endurance exercise is intriguing, too [110].

Overactivity of AhR appears to be a common feature of many inflammation-associated disorders, including CVDs. The literature contains new and intriguing findings and evidence implying the good potential of AhR targeting for the modulation of the immune response in such diseases.

The complex biology of AhR and its impact on diseases suggest that it is important to properly investigate the full set of ligand–receptor interactions of AhR and the mechanisms underlying its downstream signaling pathway. Today, additional theoretical knowledge is still needed about the possible relations of AhR with the KP. This is because situations are possible when, in response to an aberration in the production of KP metabolites, AhR signal transduction that depends on its ligands KYN or KYNA is disturbed; this may be an important mechanism regulating inflammation during disease progression. Effects of KYN and KYNA on the AhR signaling cascade require in-depth analysis to avoid unexpected adverse effects. It is also necessary to take into consideration the complexity possibly associated with cellular and tissue-specific disturbances affecting KP metabolites and with paracrine effects of such metabolites on biological processes dependent on AhR.
